# Political voting in the United Kingdom 2019 general election and risk of living with obesity in a nationally representative sample

**DOI:** 10.1038/s41366-024-01569-5

**Published:** 2024-06-26

**Authors:** A. J. Daley, A. K. Roalfe, S. N. Bleich

**Affiliations:** 1 Centre for Lifestyle Medicine and Behaviour, School of Sport, Exercise and Health Sciences, Loughborough University, Loughborough, LE11 3TU UK; 2 Department of Health Policy and Management, Harvard T.H. Chan School of Public Health, Boston, MA USA

**Keywords:** Weight management, Risk factors, Cardiovascular diseases

## Abstract

**Background:**

Limited evidence from the United States suggests that county/state rates of people with obesity are positively associated with voting for the Republican Party presidential candidate, although this question has not yet been studied at the individual level, and/or outside of the United States, where the health and political systems are very different in other countries.

**Objectives:**

Using individual level data, assess differences in rates of people with obesity according to political voting in the United Kingdom 2019 general election, and examine whether people living in constituencies won by Members of Parliament (MPs) from the Conservative Party were more likely to be living with obesity than those living in constituencies won by MPs from other parties.

**Methods:**

Data was obtained by the Ipsos KnowledgePanel where panellists are recruited via a random probability unclustered address-based sampling method. 4000/14,016 panellists were randomly invited to provide data on socio-demographics, health outcomes, voting behaviour and height/weight.

**Results:**

2668/4000 (67%) of invitees provided data, 95/2668 (3.5%) were not eligible to vote, with the remaining 2573 (96.5%) included. Conservative Party voters were more likely to be living with obesity than those who voted Labour (OR:1.42 95% CI (1.01–1.99)) or Liberal Democrats (1.54 95% CI (1.00–2.37)). Conservative Party voters on average had significantly higher BMI scores than those voting Labour and Liberal Democrats; BMI mean difference 0.88 points (95% CI: 0.16–1.61) between Conservative and Labour voters, and 1.04 points (95% CI: 0.07–2.02) between Conservatives and Liberal Democrats voters. There was no evidence participants living in constituencies won by Conservative MPs were more likely to be living with obesity than constituencies won by other party MPs.

**Conclusion:**

Governments and public health agencies may need to focus on the political affiliation of the public when developing strategies to reduce the number of people with obesity.

## Introduction

Health policy and health disparities are increasingly prominent manifesto subjects for political parties around the world, with the number of people living with obesity and associated diseases often taking a central focus. Ample research has documented the seriousness of the high number of people living with overweight as a public health problem and examined differences in rates of people with obesity by characteristics such as self-reported gender, ethnicity and socio-economic status [1–3]. Evidence also shows an association between doctors’ political affiliation and their approach to the management of those living with obesity [4]. However, little research has explored the relationship between rates of people with obesity and individual political affiliation. It is reasonable that the two may be correlated given prior evidence suggesting that voting on the political right is associated with higher rates of mortality and morbidity, than voting for the political left [5–7].

Evidence from the United States (US) has indicated that higher rates of people with obesity at the geographical county/state level (political environment) are associated with voting for the Republican Party presidential candidate [7–11]. However, this relationship is yet to be studied at an individual voter level, which is better able to control for important confounding variables and allows for more granular investigation than geographical based analyses. Additionally, the previous US election studies have used simple dichotomous comparisons of people with obesity between Republican and Democrats governed counties, but political voting in other countries is more nuanced and diverse, with several parties receiving varying amounts of electoral support. Given that the political, economic and health care systems in the US are very different to those in other countries, including the United Kingdom (UK), research from the US may have limited generalisability.

Using individual level data from a nationally representative sample in the UK, we aimed to extend and improve upon previous research and answer two questions: what are the differences in rates of people living with obesity, BMI score and proportions of BMI category according to political voting at the UK 2019 general election across multiple political parties; and are people who live in constituencies won by Members of Parliament (MPs) from the Conservative Party at the UK 2019 general election more likely to be living with obesity than those who live in constituencies won by MPs from other UK political parties (which provides the closest analysis to prior US county/state level studies) [7–11]. The answer to these questions could offer helpful insights to direct public health strategies to reduce and/or prevent the number of people with obesity in the population.

## Materials, subjects and methods

### Participant recruitment and sample

This study is based on secondary analysis from a prior study where the aim was to understand the views of the public about lifestyle behaviours [12]. Favourable ethical opinion for data collection was granted by the Loughborough University Ethical Committee for Human Participants (reference: 17722). All methods were performed in accordance with the relevant research ethics guidelines and regulations. Written informed consent was obtained from participants when they agreed to take part in the primary study. This study uses data from a nationally representative sample of adults from across the UK recruited via the UK Ipsos KnowledgePanel. Panellists are recruited using a random probability unclustered address-based sampling method where every household in the UK has a known chance of being selected to join the panel [13]. Letters are sent to selected addresses in the UK inviting occupants to become members of the panel. Up to two household occupants are permitted to join the panel. If an occupant does not have access to the internet, they can register to the KnowledgePanel by post or telephone, and are given a free tablet, an email address, and access to the internet so that they are able to complete surveys online. These inclusive methodological approaches not only improve population coverage, but also provide a more effective means for recruiting hard-to-reach members of the public.

At the time of joining the KnowledgePanel, panellists completed a general online administered informed consent process. As the KnowledgePanel is a random probability survey, panel invited samples are stratified when conducting waves to account for any profile skews within the panel. At the time of this research the KnowledgePanel contained 14,016 available panellists. Of these, 4000 were selected at random and invited to take part in the primary study on which this secondary study is based. The sample was stratified by region and education. The sample drawn was then reviewed against population statistics on ethnicity, index of multiple deprivation (IMD) [14], urbanity of home postcode (rural vs urban), age and self-reported gender. IMD contains seven domains of deprivation: income, employment, education, health, crime, barriers to housing and services; and living environment. The sample size was fixed hence no formal sample size calculations were made, however the sample size is large enough to satisfy the general rules of thumb of two participants per variable and 10 events per variable required for adequate estimation of linear and logistic regression coefficients respectively [15].

### Study survey

Data was collected between 24–30 June 2021. On joining the KnowledgePanel, panellists were asked to complete a core survey profile, which included questions about age, self-reported gender, ethnicity, income, country of residence, postcode, highest level of education and the political party voted for at the 2019 UK general election. Data for self-reported height and weight were collected as part of a separate parallel survey. Postcode was used to calculate IMD quintile and to classify the political party that won the constituency where participants lived at the 2019 general election. All data received by the research team were de-identified.

### Patient and public involvement

No patients or members of the public were involved in the planning or design of this study.

### Analysis

To ensure the findings were representative of the UK population, the below weighting specification was applied to the data in line with the target sample profile. As only two members per UK household are allowed to register with the KnowledgePanel, a design weight to correct for unequal probabilities of selection of household members was employed. Calibration weights were applied using the latest population statistics relevant to the surveyed UK population. England and Wales, Scotland and Northern Ireland were each weighted separately while an additional weight was created for the UK to account for any over or under sampling within each of these countries. Two sets of calibration weights were applied. Calibration weighting was applied using region and an interlocked variable of self-reported gender by age (both use Office of National Statistics (ONS) 2020 mid-year population estimates as the weighting target [16]. Demographic weights were then applied to correct for imbalances in the achieved sample and the data weighted on education, ethnicity, IMD (quintiles), and number of adults in the household. Estimates from the ONS 2020 mid-year population estimates and the Annual Population Survey were used as the weighting target [16]. Statistics are reported as unweighted frequencies and weighted percentages to maximize transparency, unless indicated otherwise as weighted results.

The political party categories of interest at the 2019 UK general election were Conservative, Labour, and the Liberal Democrats as these typically share the largest proportion of electoral support in the UK [17]. A small number of participants voted for other political parties (i.e., Scottish National Party, Brexit Party, Plaid Cymru, Green Party, UK Independent Party, British National Party, Democratic Unionist, Ulster Unionist, Sinn Féin, People Before Profit & Alliance). These were included in the statistical modelling as a combined group, and whilst it was important for these data to remain in the dataset for analysis purposes, their comparison with other political parties were not of interest and not reported. Analyses for the political party of participants’ constituency Member of Parliament (MPs) and living with obesity outcomes compared participants who voted for the Conservative, Labour and the Scottish National (SNP) parties as there were more voters living in constituencies won by the SNP than for the Liberal Democrats Party, the Liberal Democrats Party being included in the combined group for this analysis.

Linear regression models were used to explore the association between political party affiliation/voting and BMI score in unadjusted and adjusted analyses. Unadjusted models included political party voting as the only independent variable; adjusted models also included age group, self-reported gender, IMD quintile and country of residence (England, other). Age was treated as a categorical variable to allow for its non-linear relationship with BMI.

Logistic regression (unadjusted and adjusted models) was used to explore the differences between political party voting and BMI category in two different ways: people with obesity versus people without obesity, as well as using the BMI categories (underweight, healthy weight, people with overweight, people with obesity) (ordinal logistic) [18]. Similar models were used to investigate whether participants who live in constituencies won by Conservative Party MPs at the 2019 general election were more likely to have a higher BMI score and to be people with obesity than participants who live in constituencies won by MPs from other political parties. Model performance included R^2^ (linear regression models); pseudo R^2^ (logistic models); and variance inflation factor (VIF), where VIF > 5 was considered severe multicollinearity. Proportional odds assumptions were tested using the Brant test. Two-sided *p* values < 0.05 were considered statistically significant. Primary analysis was performed on complete cases with multiple imputation by chained equations (100 imputations) included as a sensitivity analysis using Stata version 17. Combined calibration and demographic weighting was performed using the SVYSET command in Stata.

## Results

### Participants and demographics

Of 4000 individuals invited to participate in the primary study, 2668 surveys were completed (67%). Of these, 95/2,668 (3.5%) were not eligible to vote in the 2019 UK general election and were excluded. Of the remaining 2573 participants, 33.3% of participants voted Conservative at the 2019 general election, 25.1% Labour, 10.6% Liberal Democrats, and 10.6% voted for a range of other political parties, with missing data for 20.7% (Table 1). A total of 14.1% (95% CI 12.2–16.3) of those reporting their political affiliation party had missing BMI data and there was no association between missing BMI and political affiliation, (weighted chisq, *p* = 0.46). A small percentage of the overall variation (R^2^ ~ 7%) in BMI was explained by the models. No evidence of multicollinearity was observed (VIF < 2.5). The proportional odds assumption for the ordinal logistic regression analyses was met (Brant test, *p* > 0.05).

**Table 1:** Participant characteristics.

	UK 2019 general election					
Political parties	Conservative	Labour	Liberal democrats	Other	Missing	Total
	*N* (wtd %)	*N* (wtd %)	*N* (wtd %)	*N* (wtd %)	*N* (wtd %)	*N* (wtd %)
Overall	1000 (33.3)	562 (25.1)	278 (10.6)	256 (10.6)	465 (20.7)	2573
Age						
16–24	11 (2.9)	26 (12.3)	7 (8.2)	12 (12.6)	35 (18.0)	91 (10.0)
25–34	36 (7.5)	80 (24.7)	20 (14.2)	30 (21.3)	58 (18.9)	224 (16.4)
35–44	77 (11.1)	101 (21.3)	32 (14.1)	36 (17.5)	59 (15.7)	305 (15.6)
45–54	172 (18.8)	107 (16.1)	52 (16.5)	47 (15.6)	96 (18.1)	474 (17.4)
55–64	273 (19.9)	122 (11.5)	70 (16.3)	77 (18.3)	123 (15.0)	665 (16.2)
65–74	303 (21.3)	97 (9.2)	68 (13.0)	55 (10.5)	70 (7.4)	593 (13.4)
75+	128 (18.6)	29 (4.5)	29 (17.7)	11 (4.2)	24 (6.8)	221 (11.1)
Gender (self reported)						
Male	521 (50.3)	275 (50.1)	140 (48.9)	136 (53.4)	190 (39.0)	1262 (48.1)
Female	475 (49.3)	281 (48.8)	137 (50.9)	131 (46.4)	272 (60.3)	1296 (51.3)
Missing	4 (0.4)	6 (1.1)	1 (0.2)	1 (0.2)	3 (0.7)	15 (0.6)
Ethnicity						
White	980 (96.9)	522 (85.3)	269 (92.3)	255 (90.6)	434 (86.2)	2460 (90.6)
Ethnic minorities	12 (2.2)	39 (14.7)	9 (7.7)	10 (7.7)	21 (11.3)	91 (8.4)
Missing	8 (0.9)	1 (0.07)	0 (0)	3 (1.7)	10 (2.5)	22 (1.0)
Country						
England	897 (90.3)	491 (87.8)	250 (90.7)	124 (46.9)	393 (85.2)	2155 (84.1)
Scotland	53 (4.4)	31 (4.6)	23 (7.2)	87 (32.3)	33 (5.7)	227 (8.0)
Wales	47 (5.1)	38 (7.2)	4 (1.6)	13 (4.0)	19 (5.2)	121 (5.1)
Northern Ireland	3 (0.2)	2 (0.4)	1 (0.5)	44 (16.9)	20 (4.0)	70 (2.8)
Marital status						
Single/widowed/divorced /separated	327 (35.6)	271 (53.9)	100 (40.8)	131 (50.3)	226 (56.2)	1055 (46.5)
Married/civil partnership	668 (63.8)	283 (45.0)	176 (58.4)	133 (46.1)	223 (40.6)	1483 (51.8)
Missing	5 (0.7)	8 (1.2)	2 (0.8)	4 (3.6)	16 (3.3)	35 (1.7)
Annual income (£)						
<£15,599	70 (6.1)	58 (10.1)	26 (7.7)	39 (12.4)	66 (13.1)	259 (9.4)
£15,600 to £25,999	157 (15.5)	92 (14.1)	38 (9.0)	45 (13.5)	61 (13.7)	393 (13.9)
£26,000 to £51,999	337 (32.3)	196 (33.0)	78(28.6)	78 (26.0)	104 (20.8)	793 (29.0)
£52,000 to £150,000+	266 (27.9)	138 (25.6)	105 (40.8)	65 (29.1)	75 (15.8)	649 (26.3)
Missing	170 (18.2)	78 (17.2)	31 (14.0)	41 (19.0)	159 (36.7)	479 (21.4)
Employment status						
Working	473 (49.0)	335 (63.0)	156 (58.7)	154 (61.4)	257 (52.7)	1375 (55.6)
Unemployed	13 (1.3)	21 (4.8)	5 (2.0)	3 (2.5)	22 (5.1)	64 (3.2)
Not working retired	432 (39.1)	140 (15.2)	104 (33.0)	77 (16.9)	101 (15.0)	854 (25.1)
Other	80 (10.4)	65 (16.7)	13 (6.3)	33 (17.9)	79 (25.2)	270 (15.4)
Missing	2 (0.3)	1 (0.4)	0 (0)	1 (1.4)	6 (2.1)	10 (0.7)
IMD quintile						
1 most deprived	92 (12.0)	116 (26.5)	17 (7.6)	38 (18.5)	103 (23.9)	366 (18.3)
2	176 (18.0)	104 (22.2)	29 (11.5)	53 (23.2)	90 (22.8)	452 (19.9)
3	212 (21.3)	113 (18.0)	50 (21.1)	71 (22.3)	95 (19.2)	541 (20.1)
4	243 (24.0)	117 (17.8)	75 (26.4)	58 (22.9)	83 (15.3)	576 (20.8)
5 least deprived	277 (24.7)	112 (15.5)	107 (33.4)	48 (13.2)	94 (18.8)	638 (20.9)
Education						
Degree level	205 (19.6)	252 (43.2)	139 (46.7)	91 (34.7)	98 (17.5)	785 (29.6)
Missing	10 (0.7)	1 (0.1)	2 (0.6)	1 (1.4)	9 (1.8)	23 (0.8)
BMI category						
Underweight	14 (1.1)	10 (3.3)	4 (1.2)	1 (0.2)	4 (0.9)	33 (1.5)
Healthy weight	333 (32.9)	204 (35.7)	126 (47.3)	91 (32.7)	155 (31.5)	909 (34.9)
People with overweight	329 (31.1)	189 (29.5)	81 (27.8)	94 (34.8)	126 (25.4)	819 (29.6)
People with obesity	203 (20.5)	90 (16.4)	40 (13.7)	51 (17.5)	82 (17.9)	466 (17.9)
Missing	121 (14.3)	69 (15.2)	27 (10.0)	31 (14.8)	98 (24.2)	346 (16.2)

*wtd* weighted

### Political voting and likelihood of people living with obesity

Conservative Party voters were more likely to be people living with obesity than those who voted Labour (AdjOR: 1.42 (1.01–1.99)), or Liberal Democrats (AdjOR: 1.54 (1.00–2.37)) (Table 2). There were no significant differences in the odds of people living with obesity between Labour and Liberal Democrats voters (Table 3).

**Table 2:** Political voting at the 2019 UK general election, people with obesity and BMI categories.

	Political party			Comparison of political parties							
Outcome	Conservative	Labour	Liberal democrats	Conservative vs labour				Conservative vs liberal democrats			
	*n* (wtd %)	*n* (wtd %)	*n* (wtd %)	Odds ratio (95% CI)	*P* value	Adjusted odds ratio (95% CI)	*P* value	Odds ratio (95% CI)	*P* value	Adjusted odds ratio (95% CI)	*P* value
People with obesity category											
People with obesity	203 (23.9)	90 (19.3)	40 (15.2)	1.32 (0.95–1.83)	0.10	1.42 (1.01–1.99)	0.042	1.75 (1.13–2.70)	0.01	1.54 (1.00–2.37)	0.049
People without obesity (reference category)	676 (76.1)	403 (81.7)	211 (84.8)								
BMI category											
People with obesity	203 (23.9)	90 (19.3)	40 (15.2)	1.34^a^ (1.03–1.75)	0.03	1.32^a^ (1.01–1.72)	0.039	1.73^a^ (1.26–2.38)	0.001	1.47^a^ (1.07–2.02)	0.017
People with overweight	329 (36.3)	189 (34.8)	81 (30.9)								
Healthy weight	333 (38.4)	204 (42.1)	126 (52.6)								
Underweight	14 (1.3)	10 (3.8)	4 (1.3)								

*N* = 1860 for unadjusted models; *N* = 1844 for adjusted models.

Wtd weighted.

^a^Proportional odds of an increase in outcome category between political parties.

**Table 3:** Political voting at the 2019 UK general election, people with obesity and BMI categories (Labour vs Liberal Democrats).

	Political party			Comparison of political parties			
Outcome	Conservative	Labour	Liberal democrat	Labour vs liberal democrats			
	*n* (wtd %)	*n* (wtd %)	*n* (wtd %)	Odds ratio (95% CI)	*P* value	Adjusted odds ratio (95% CI)	*P* value
People with obesity category							
People with obesity	203 (23.9)	90 (19.3)	40 (15.2)	1.33 (0.82–2.15)	0.25	1.08 (0.67–1.75)	0.74
People without obesity (reference category)	676 (76.1)	403 (80.7)	211 (84.8)				
BMI category							
People with obesity	203 (23.9)	90 (19.3)	40 (15.2)	1.29^a^ (0.90–1.86)	0.17	1.11^a^(0.78–1.60)	0.56
People with overweight	329 (36.3)	189 (34.8)	81 (30.9)				
Healthy weight	333 (38.4)	204 (42.1)	126 (52.6)				
Underweight	14 (1.3)	10 (3.8)	4 (1.3)				

*N* = 1860 for unadjusted models, *N* = 1844 for adjusted models.

*Wtd* weighted.

^a^Proportional odds of an increase in outcome category between political parties.

### Political voting and BMI

Conservative Party voters had a significantly higher BMI score than Labour or Liberal Democrats voters with unadjusted means (SE) of 27.18 (0.22), 26.34 (0.31) and 25.73 (0.46) respectively and mean differences of 0.84 units (95% CI 0.08–1.59) and 1.45 (0.44–2.46). Multiple linear regression analyses demonstrated an adjusted mean difference in BMI of 0.88 units (95% CI: 0.16–1.61) between Conservative and Labour voters and 1.04 units (95% CI: 0.07–2.02) between Conservative and Liberal Democrats voters (Supplementary Table 1). A significant association was recorded between political affiliation and BMI category (underweight, healthy, people with overweight and people with obesity) in unadjusted and adjusted models. The odds of being in a higher BMI category was 1.32 (1.01–1.72) times greater for Conservative than Labour Party voters and 1.47 (1.07–2.02) times greater for Conservative than Liberal Democrats Party voters in adjusted models (Table 2).

### Political party of constituency MPs and rates of people living with obesity

At the 2019 general election 57.9% (*n* = 1622) of participants lived in constituencies won by Conservative MPs, with 30.2% (*n* = 629) in Labour and 6.6% (*n* = 182) in Scottish National Party (SNP) won seats. There was no significant difference in the prevalence of people living with obesity, BMI category or BMI score between the political parties of participants’ constituency MP (Conservative vs Labour, Conservative vs SNP, Labour vs SNP). See Tables 4, 5 and Supplementary Table 2.

**Table 4:** Winning constituency MP at the 2019 UK general election, people with obesity and BMI categories.

	Winning political party			Comparison of winning political parties							
Outcome	Conservative	Labour	SNP	Conservative vs labour				Conservative vs SNP			
	*n* (wtd %)	*n* (wtd %)	*n* (wtd %)	Odds ratio (95% CI)	*P* value	Adjusted odds ratio (95% CI)	*P* value	Odds ratio (95% CI)	*P* value	Adjusted odds ratio (95% CI)	*P* value
People with obesity category											
People with obesity	293 (21.0)	118 (22.5)	32 (20.3)	0.91 (0.68–1.22	0.55	1.08 (0.78–1.47)	0.65	1.04 (0.65–1.67)	0.87	1.34 (0.68–2.63)	0.39
People without obesity (reference category)	1131 (79.0)	404 (77.5)	125 (79.7)								
BMI category											
People with obesity	293 (21.0)	118 (22.5)	32 (20.3)	0.96^a^ (0.75–1.22)	0.73	1.06^a^ (0.82–1.37)	0.65	0.93^a^ (0.67–1.29	0.66	1.31^a^ (0.79–2.18)	0.30
People with overweight	523 (35.1)	194 (35.0)	59 (39.1)								
Healthy weight	588 (42.6)	202 (39.5)	163 (39.2)								
Underweight	20 (1.3)	8 (3.0)	3 (1.4)								

*N* = 2227 for unadjusted models; *N* = 2205 for adjusted models.

*Wtd* weighted.

^a^Proportional odds of an increase in outcome category between political parties.

**Table 5:** Winning constituency MP at the 2019 UK general election, people with obesity category and BMI categories.

	Winning political party			Comparison of winning political parties			
Outcome	Conservative	Labour	SNP	Labour vs SNP			
	*n* (wtd %)	*n* (wtd %)	*n* (wtd %)	Odds ratio (95% CI)	*P* value	Adjusted odds ratio (95% CI)	*P* value
People with obesity category							
People with obesity	293 (21.0)	118 (22.5)	32 (20.3)	1.14 (0.68–1.89)	0.62	1.25 (0.64–2.45)	0.52
People without obesity (reference category)	1131 (79.0)	404 (77.5)	125 (79.7)				
BMI category							
People with obesity	293 (21.0)	118 (22.5)	32 (20.3)	0.97^a^ (0.67–1.41)	0.87	1.23^a^(0.74–2.07)	0.43
People with overweight	523 (35.1)	194 (35.0)	59 (39.1)				
Healthy weight	588 (42.6)	202 (39.5)	163 (39.2)				
Underweight	20 (1.3)	8 (3.0)	3 (1.4)				

*N* = 2227 for unadjusted models; *N* = 2205 for adjusted models.

*Wtd* weighted.

^a^Proportional odds of an increase in BMI between political parties.

### Sensitivity analysis

A total of 28.3% and 14.3% of data were missing for the political voting models and winning MP models respectively. Multiple imputation, assuming data were missing at random, produced similar results to the complete case analyses (Supplementary Tables 3 and 4).

## Discussion

Health remains an important political issue and a top priority for voters. In this nationally representative sample, after adjusting for age group, self-reported gender, ethnicity, indicators of deprivation and country, voters for the Conservative Party had a higher BMI score (~1 unit) and were more likely to be living with obesity (between 42–54% higher odds) than Labour and Liberal Democrats Party voters. This finding is notable as living with obesity increases the risk of morbidity, dying prematurely and has substantial economic consequences for health care services across the world [19]. Given that almost 14 million people voted for the Conservative Party at the 2019 UK general election, the largest majority since 1987 [17], governments and public health agencies may benefit from focusing on the political affiliation of the public when developing strategies to prevent and reduce the number of people with obesity.

### Comparison with the literature

A few studies conducted in the US have evaluated the association between political voting and the risk of living with obesity from a county/state geographical perspective [7–11]. However, the political environment where people live is likely to have a more distal impact on the number of people with obesity, than individuals’ health behaviour decisions. Consistent with the previous US studies [7–11], but using individual level data, this UK based study found that Conservative Party voters had a significantly higher BMI score (~1 BMI unit) and were more likely to be people with obesity. This finding is also consistent with evidence from the US reporting that mortality rates have decreased (2001–2019) by 22% in Democrats governed counties and by only 11% in Republican governed counties, and that two of the largest contributors to this rising gap in mortality were heart disease and cancer [5].

The political affiliation of the public could play a role in the adoption of public health polices and engagement with health-protective behaviours. Republican/Conservative Party voters may be less likely to support or comply with policies to reduce/prevent the probability of living with obesity. For example, research has reported that Republicans (Conservatives) consume fewer servings and varieties of fruit and vegetables, more high fat and processed foods, more meat, and are less likely to participate in exercise than Democrat voters, behaviours associated with increasing the risk of people living with obesity [20, 21]. Further research is required to understand the factors that are driving these higher rates of people with overweight/obesity in voters who affiliate with the political right.

Unlike the US studies, we did not find that participants living in constituencies won by Conservative Party MPs were more likely to be people with obesity, than for constituencies won by the Labour and Liberal Democrats parties. This disparity between the US studies and our UK study may be explained by differences in the political systems in the countries. US politicians have more local decision-making power than MPs in the UK, with many of the policies to prevent or reduce the rates of people with obesity established at the state or local level in the US (e.g., taxes to reduce consumption of sugary beverages and food warning labels). Evidence suggests that US Republican politicians view the resolution of people living with obesity as more of a personal responsibility by individuals, than do politicians from the Democrats Party, and they are less likely to drive forward policies/laws to reduce the number of people with obesity through preventative healthcare measures [22], potentially leading to high rates of people with obesity in residents of Republican (conservative) governed states. In the UK, health policies are broadly implemented at a national government level, not by local individual constituency MPs.

### Implications

The clinical relevance of the ~1 unit difference in BMI score between voters for the Conservative Party and other political parties translates into 26 and 28 fewer cases of chronic disease per 1000 men and women, respectively [23]. Campaigns may need to be targeted at supporters of the Conservative Party (and equivalent political parties in other countries), including during times of local and national elections. Results from this study raise an interesting and sensitive dilemma for health policy. It has been previously reported that individuals with conservative political views tend to have better health than those who hold more liberal attitudes because conservatives place greater value on personal responsibility [24], leading to greater adoption of health-related behaviours and better health outcomes. However, this study does not support this assertion as far as living with obesity is concerned. Additionally, Conservative Party voters have traditionally been more affluent, therefore may have better access to resources to manage their own weight, relative to voters for alternative political parties [25, 26]. That said, data from the 2019 general election indicated that the Conservative Party was now more popular with those who have low incomes, compared to those with high incomes [27]. Moreover, for the first time in recorded history, the Conservative Party performed better in a general election than the Labour Party among voters who have low-income.

### Strengths and weakness

To our knowledge this is the first study to be published on the association between political affiliation and rates of living with obesity and weight status using individual level data collected outside of the US. Until now it has been difficult to investigate direct relationships between health behaviours/outcomes and political affiliation in the UK because health surveys do not routinely question respondents about their political affiliation. The use of a large national panel and a high response rate should reduce any possible concerns about response bias and survey weights were applied in the analyses to account for any response differences. A strength of this study is the use of a KnowledgePanel, where panellists who do not have an easy way to access to the internet (typically people on the lowest incomes), are given resources to ensure that a lack of access is not a barrier to participation in research. This methodological inclusive approach is critical to the integrity of the findings from studies that are focused on answering questions about relationship(s) between politics and health, particularly those which are intended to be nationally representative. Specifically, this approach facilitates representation of participants from of across the political spectrum. Because our analyses were based on individual level data, we were able to adjust for variables that were not included in prior studies that were based on aggregated/geographical area (county and state) data.

This study is observational and used data from one general election so causality cannot be assumed. Political affiliation is a private and sensitive topic and people can be reluctant to disclose this information, and not all adults will vote in a general election, contributing to missing data. Height and weight (for calculation of BMI) were self-reported and these data may be subject to underreporting, although if true this would suggest findings represent a better scenario than is actually the case. Missing data were not related to political affiliation providing reassurance that data were not disproportionate to a particular political party. Multiple imputation of missing data also provides some evidence of the robustness of our results. We have controlled for a range of variables in our analyses but cannot rule out that other unknown factors impacted the results. We did not find that participants living in constituencies won by Conservative Party MPs were more likely to be living with obesity than for constituencies won by other political parties, although this may be because the sample size was not sufficiently large to do so.

## Conclusion

Participants who voted for the Conservative Political Party at the UK 2019 general election had a higher BMI and were more likely to be people with obesity than those voting for other political parties. Given the well documented association between living with obesity and risk for morbidity and mortality and the size of the population electorate, public health messages and interventions to curb the number of people with excess body weight may benefit from taking political orientation into consideration.

## Supplementary information

Supplementary file of tables

## Data Availability

The datasets generated during and/or analysed during the current study are available from the corresponding author on reasonable request.
