# Supplementary material for: Political voting in the United Kingdom 2019 general election and risk of living with obesity in a nationally representative sample
Source: Int J Obes (Lond). 2024 Jun 26;48(10):1430–7. doi: 10.1038/s41366-024-01569-5 (PMC11420055; doi:10.1038/s41366-024-01569-5)
Supplement: Supplementary file 1 — Supplementary file of tables [file 41366_2024_1569_MOESM1_ESM.docx]

**Supplementary Table 1: Association of political voting at the 2019 UK general election with BMI, people with obesity and BMI categories**

| Model parameters | Linear model for BMI score | | Logistic model for people with obesity | | Ordinal logistic model for BMI category | |
| --- | --- | --- | --- | --- | --- | --- |
|  | Coefficient (95% CI) | P value | Adjusted Odds ratio (95% CI) | P value | Adjusted Odds ratio* (95% CI) | P value |
| **Political voting** |  |  |  |  |  |  |
| Conservative | 0.88 (0.16 to 1.61) | 0.016 | 1.42 (1.01 to 1.99) | 0.04 | 1.32 (1.01 to 1.72) | 0.039 |
| Liberal Democrats | -0.16 (-1.24 to 0.93) | 0.77 | 0.92 (0.57 to 1.49) | 0.74 | 0.90 (0.62 to 1.29) | 0.56 |
| Other | 0.54 (-0.70 to 1.79) | 0.39 | 1.05 (0.62 to 1.80) | 0.84 | 1.21 (0.81 to 1.81) | 0.35 |
| Labour (reference category) |  |  |  |  |  |  |
| **Age category** |  |  |  |  |  |  |
| 16-24 | -0.90 (-2.43 to 0.62) | 0.25 | 1.13 (0.37 to 3.46) | 0.83 | 0.46 (0.20 to 1.02) | 0.056 |
| 25-34 | 1.76 (0.58 to 2.95) | 0.004 | 2.88 (1.44 to 5.77) | 0.003 | 1.76 (1.12 to 2.75) | 0.014 |
| 35-44 | 1.84 (0.66 to 3.03) | 0.002 | 3.15 (1.67 to 5.96) | <0.001 | 1.52 (1.00 to 2.31) | 0.051 |
| 45-54 | 2.63 (1.70 to 3.57) | <0.001 | 4.59 (2.57 to 8.21) | <0.001 | 2.51 (1.75 to 3.59) | <0.001 |
| 55-64 | 1.74 (0.90 to 2.58) | <0.001 | 2.60 (1.46 to 4.64) | 0.001 | 1.78 (1.28 to 2.47) | 0.001 |
| 65-74 | 1.25 (0.43 to 2.08) | 0.003 | 2.36 (1.34 to 4.17) | 0.003 | 1.49 (1.08 to 2.06) | 0.015 |
| 75+ (reference category) |  |  |  |  |  |  |
| **Gender (self reported)** |  |  |  |  |  |  |
| Male | 0.84 (0.23 to 1.44) | 0.007 | 1.21 (0.92 to 1.60) | 0.17 | 1.47 (1.19 to 1.82) | <0.001 |
| Female (reference category) |  |  |  |  |  |  |
|  |  |  |  |  |  |  |
| **Ethnicity** |  |  |  |  |  |  |
| White | 1.33 (-0.06 to 2.73) | 0.061 | 2.04 (0.86 to 4.82) | 0.10 | 1.77 (0.99 to 3.16) | 0.052 |
| Ethnic minorities (reference category) |  |  |  |  |  |  |
|  |  |  |  |  |  |  |
| **IMD quintile** |  |  |  |  |  |  |
| 1 most deprived | 1.57 (0.59 to 2.55) | 0.002 | 2.22 (1.39 to 3.53) | 0.001 | 1.80 (1.23 to 2.63) | 0.003 |
| 2 | 1.96 (0.92 to 3.01) | <0.001 | 2.35 (1.52 to 3.62) | <0.001 | 1.65 (1.17 to 2.33) | 0.004 |
| 3 | 1.19 (0.42 to 1.97) | 0.003 | 1.86 (1.23 to 2.80) | 0.003 | 1.52 (1.13 to 2.03) | 0.005 |
| 4 | 1.04 (0.33 to 1.74) | 0.004 | 1.94 (1.30 to 2.90) | 0.001 | 1.42 (1.07 to 1.89) | 0.014 |
| 5 least deprived (reference category) | - |  |  |  |  |  |
|  |  |  |  |  |  |  |
| **Country** |  |  |  |  |  |  |
| England | -0.26 (-1.16 to 0.64) | 0.57 | 0.99 (0.65 to 1.49) | 0.94 | 0.87 (0.63 to 1.20) | 0.39 |
| Other (reference category) |  |  |  |  |  |  |
|  |  |  |  |  |  |  |
| Intercept | 22.3 (20.3 to 24.2) | <0.001 | 0.02 (0.007 to 0.07) | <0.001 |  |  |
| Cutpoint 1 |  |  |  |  | -2.67 (-3.57 to -1.77) |  |
| Cutpoint 2 |  |  |  |  | 1.24 (0.48 to 2.00) |  |
| Cutpoint 3 |  |  |  |  | 2.95 (2.18 to 3.71) |  |
| R^2^ | 0.068 |  | 0.054 |  |  |  |

*proportional odds of an increase in outcome category between political parties. N=1,844

**Supplementary Table 2: Association of winning constituency MP at the 2019 UK general election with BMI, people with obesity and BMI categories**

| Model parameters | Linear model for BMI score | | Logistic model for people with obesity | | Ordinal logistic model for BMI category | |
| --- | --- | --- | --- | --- | --- | --- |
|  | Coefficient (95% CI) | P value | Adjusted Odds ratio (95% CI) | P value | Adjusted Odds ratio* (95% CI) | P value |
| **Winning Political Party** |  |  |  |  |  |  |
| Conservative | 0.06 (-0.69 to 0.82) | 0.87 | 1.08 (0.78 to 1.47) | 0.65 | 1.06 (0.82 to 1.37) | 0.65 |
| Scottish National Party | -0.42 (-1.78 to 0.94) | 0.55 | 0.80 (0.41 to 1.57) | 0.52 | 0.81 (0.48 to 1.36) | 0.43 |
| Other | -0.14 (-1.46 to 1.18) | 0.83 | 0.96 (0.49 to 1.89) | 0.92 | 0.91 (0.54 to 1.54) | 0.72 |
| Labour (reference category) |  |  |  |  |  |  |
| **Age category** |  |  |  |  |  |  |
| 16-24 | -0.86 (-2.19 to 0.48) | 0.21 | 0.87 (0.32 to 2.33) | 0.78 | 0.64 (0.33 to 1.26) | 0.20 |
| 25-34 | 1.90 (0.78 to 3.02) | 0.001 | 2.73 (1.43 to 5.22) | 0.002 | 1.77 (1.17 to 2.69) | 0.007 |
| 35-44 | 1.83 (0.74 to 2.92) | <0.001 | 2.86 (1.53 to 5.35) | 0.001 | 1.60 (1.08 to 2.37) | 0.018 |
| 45-54 | 2.56 (1.70 to 3.42) | <0.001 | 3.94 (2.20 to 7.07) | <0.001 | 2.48 (1.76 to 3.49) | <0.001 |
| 55-64 | 1.84 (1.05 to 2.63) | <0.001 | 2.41 (1.35 to 4.29) | 0.003 | 1.84 (1.34 to 2.54) | <0.001 |
| 65-74 | 1.18 (0.41 to 1.95) | 0.003 | 1.92 (1.08 to 3.43) | 0.03 | 1.48 (1.08 to 2.02) | 0.014 |
| 75+ (reference category) |  |  |  |  |  |  |
| **Gender (self reported)** |  |  |  |  |  |  |
| Male | 0.66 (0.09 to 1.23) | 0.022 | 1.15 (0.89 to 1.48) | 0.28 | 1.40 (1.15 to 1.71) | 0.001 |
| Female (reference category) |  |  |  |  |  |  |
|  |  |  |  |  |  |  |
| **Ethnicity** |  |  |  |  |  |  |
| White | 1.16 (-0.14 to 2.46) | 0.08 | 1.96 (0.93 to 4.10) | 0.08 | 1.41 (0.79 to 2.53) | 0.30 |
| Ethnic minorities (reference category) |  |  |  |  |  |  |
|  |  |  |  |  |  |  |
| **IMD quintile** |  |  |  |  |  |  |
| 1 most deprived | 1.81 (0.89 to 2.74) | <0.001 | 2.34 (1.55 to 3.54) | <0.001 | 1.80 (1.28 to 2.50) | 0.001 |
| 2 | 1.70 (0.79 to 2.60) | <0.001 | 2.05 (1.38 to 3.04) | <0.001 | 1.54 (1.13 to 2.11) | 0.007 |
| 3 | 1.24 (0.54 to 1.95) | 0.001 | 2.00 (1.38 to 2.90) | <0.001 | 1.54 (1.17 to 2.01) | 0.002 |
| 4 | 0.90 (0.24 to 1.56) | 0.008 | 1.70 (1.18 to 2.45) | 0.005 | 1.36 (1.04 to 1.77) | 0.024 |
| 5 least deprived (reference category) |  |  |  |  |  |  |
|  |  |  |  |  |  |  |
| **Country** |  |  |  |  |  |  |
| England | -0.32 (-1.32 to 0.69) | 0.54 | 0.94 (0.58 to 1.53) | 0.80 | 0.81 (0.55 to 1.20) | 0.30 |
| Other (reference category) |  |  |  |  |  |  |
|  |  |  |  |  |  |  |
| Intercept | 23.1 (22.2 to 24.9) | <0.001 | 0.03 (0.01 to 1.53) | <0.001 |  |  |
| Cutpoint 1 |  |  |  |  | -2.85 (-3.71 to 1.99) |  |
| Cutpoint 2 |  |  |  |  | 1.05 (0.32 to 1.79) |  |
| Cutpoint 3 |  |  |  |  | 2.71 (1.97 to 3.44) |  |
| R^2^ | 0.058 |  | 0.049 |  |  |  |

*proportional odds of an increase in outcome category between political parties. N=2,205.

**Supplementary Table 3: Multiple imputation results for political voting at the 2019 UK general election with BMI, people with obesity and BMI categories**

|  | **Comparison of political parties** | | | | | |
| --- | --- | --- | --- | --- | --- | --- |
|  | Conservative vs Labour | | Conservative vs Liberal Democrats | | Labour vs Liberal Democrats | |
| **BMI** | Difference in Adjusted Means (95% CI) | P value | Difference in Adjusted Means (95% CI) | P value | Difference in Adjusted Means (95% CI) | P value |
|  | 0.84 (0.04 to 1.64) | 0.039 | 1.18 (0.22 to 2.14) | 0.016 | 0.33 (-0.71 to 1.38) | 0.53 |
|  |  |  |  |  |  |  |
| **People with obesity category** | Adjusted odds ratio (95% CI) | P value | Adjusted odds ratio (95% CI) | P value | Adjusted odds ratio (95% CI) | P value |
| People with obesity | 1.38 (0.98 to 1.96) | 0.07 | 1.57 (0.99 to 2.50) | 0.054 | 1.14 (0.70 to 1.85) | 0.61 |
| People without obesity (reference category) |  |  |  |  |  |  |
|  |  |  |  |  |  |  |
| **BMI category** | Adjusted odds ratio* (95% CI) | P value | Adjusted odds ratio* (95% CI) | P value | Adjusted odds ratio* (95% CI) | P value |
| People with obesity | 1.29 (0.98 to 1.71) | 0.07 | 1.53 (1.10 to 2.12) | 0.011 | 1.18 (0.82 to 1.72) | 0.38 |
| People with overweight |  |  |  |  |  |  |
| Healthy weight |  |  |  |  |  |  |
| Underweight |  |  |  |  |  |  |

*proportional odds of an increase in outcome category between political parties; N=2573; 100 imputations;

**Supplementary Table 4: Multiple imputation results for winning constituency MP at the 2019 UK general election with BMI, people with obesity and BMI categories**

|  | **Comparison of winning political parties** | | | | | |
| --- | --- | --- | --- | --- | --- | --- |
|  | Conservative vs Labour | | Conservative vs SNP | | Labour vs SNP | |
| **BMI** | Difference in Adjusted Means (95% CI) | P value | Difference in Adjusted Means (95% CI) | P value | Difference in Adjusted Means (95% CI) | P value |
|  | 0.14 (-0.61 to 0.89) | 0.71 | 0.41 (-0.92 to 1.73) | 0.55 | 0.26 (-1.11 to 1.64) | 0.71 |
|  |  |  |  |  |  |  |
| **People with obesity category** | Adjusted odds ratio (95% CI) | P value | Adjusted odds ratio (95% CI) | P value | Adjusted odds ratio (95% CI) | P value |
| People with obesity | 1.08 (0.78 to 1.50) | 0.63 | 1.26 (0.65 to 2.45) | 0.50 | 1.16 (0.59 to 2.28) | 0.66 |
| People without obesity (reference category) |  |  |  |  |  |  |
|  |  |  |  |  |  |  |
| **BMI category** | Adjusted odds ratio* (95% CI) | P value | Adjusted odds ratio* (95% CI) | P value | Adjusted odds ratio* (95% CI) | P value |
| People with obesity | 1.08 (0.84 to 1.40) | 0.54 | 1.24 (0.75 to 2.04) | 0.40 | 1.14 (0.69 to 1.90) | 0.61 |
| People with overweight |  |  |  |  |  |  |
| Healthy weight |  |  |  |  |  |  |
| Underweight |  |  |  |  |  |  |

*proportional odds of an increase in outcome category between political parties; N=2573; 100 imputations;
